# The Trial-Ready Cohort for Preclinical and Prodromal Alzheimer’s Disease (TRC-PAD): Experience from the First 3 Years

**DOI:** 10.14283/jpad.2020.47

**Published:** 2020

**Authors:** S. Walter, O.G. Langford, T.B. Clanton, G.A. Jimenez-Maggiora, R. Raman, M.S. Rafii, E.J. Shaffer, R.A. Sperling, J.L. Cummings, P.S. Aisen, TRC-PAD Investigators

**Affiliations:** 1. Alzheimer’s Therapeutic Research Institute, University of Southern California, San Diego, CA, USA; 2. Center for Alzheimer Research and Treatment, Brigham and Women’s Hospital, Harvard Medical School, Boston, MA, USA; 3. Department of Brain Health, School of Integrated Health Sciences, University of Las Vegas, Nevada; Cleveland Clinic Lou Ruvo Center for Brain Health, USA

**Keywords:** Alzheimer’s disease, prevention, webstudy, remote study

## Abstract

**BACKGROUND:**

The Trial-Ready Cohort for Preclinical and Prodromal Alzheimer’s disease (TRC-PAD) aims to accelerate enrollment for Alzheimer’s disease (AD) clinical trials by remotely identifying and tracking individuals who are at high risk for developing symptoms of AD, and referring these individuals to in-person cognitive and biomarker evaluation with the purpose of engaging them in clinical trials. A risk algorithm using statistical modeling to predict brain amyloidosis will be refined as TRC-PAD advances with a maturing data set.

**OBJECTIVES:**

To provide a summary of the steps taken to build this Trial-Ready cohort (TRC) and share results of the first 3 years of enrollment into the program.

**DESIGN:**

Participants are remotely enrolled in the Alzheimer Prevention Trials (APT) Webstudy with quarterly assessments, and through an algorithm identified as potentially at high risk, referred to clinical sites for biomarker confirmation, and enrolled into the TRC.

**SETTING:**

Both an online study and in-clinic non-interventional cohort study.

**PARTICIPANTS:**

APT Webstudy participants are aged 50 or older, with an interest in participation in AD therapeutic trials. TRC participants must have a study partner, stable medical condition, and elevated brain amyloid, as measured by amyloid positron emission tomography or cerebrospinal fluid analysis. Additional risk assessments include apolipoprotein E genotyping.

**MEASUREMENTS:**

In the APT Webstudy, participants complete the Cognitive Function Index and Cogstate Brief Battery. The TRC includes the Preclinical Alzheimer’s Cognitive Composite, comprised of the Free and Cued Selective Reminding Test, the Delayed Paragraph Recall score on the Logical Memory IIa test from the Wechsler Memory Scale, the Digit-Symbol Substitution test from the Wechsler Adult Intelligence Scale-Revised, and the Mini Mental State Examination total score (1).

**RESULTS:**

During the first 3 years of this program, the APT Webstudy has 30,650 consented participants, with 23 sites approved for in person screening, 112 participants have been referred for in-clinic screening visits with eighteen enrolled to the TRC. The majority of participants consented to APT Webstudy have a family history of AD (62%), identify as Caucasian (92.5%), have over twelve years of formal education (85%), and are women (73%). Follow up rates for the first quarterly assessment were 38.2% with 29.5% completing the follow up Cogstate Battery.

**CONCLUSIONS:**

After successfully designing and implementing this program, the study team’s priority is to improve diversity of participants both in the APT Webstudy and TRC, to continue enrollment into the TRC to our target of 2,000, and to improve longitudinal retention, while beginning the process of referring TRC participants into clinical trials.

## Background

The Trial-Ready Cohort for Preclinical and Prodromal Alzheimer’s disease (TRC-PAD) program aims to accelerate enrollment into clinical trials for AD by building a cohort of biomarker-confirmed eligible participants. The first stage of the program is remote recruitment of participants to the Alzheimer Prevention Trials (APT) Webstudy (2). Participants are followed with quarterly assessments, and through an algorithm identified as potentially at high risk, and referred to clinical sites. Participants are screened for Trial-ready cohort (TRC) eligibility, involving cognitive testing, genotyping and amyloid biomarker measures, and then if eligible, enrolled and followed longitudinally until an appropriate clinical trial becomes available. Separate papers summarize the program design and implementation considerations (3), the complex informatics infrastructure (4), the algorithm to predict brain amyloidosis and risk for AD (5), and recruitment strategies (2). Here we expand on the experience of the TRC-PAD program during its initial three years.

### Study network and infrastructure

The TRC-PAD program is the result of extensive collaboration between multiple principal investigators (PIs), online National Registries, the Coordinating Center, and the network of clinical trial sites. Registries that referred participants to the APT Webstudy were the Alzheimer’s Prevention Registry (APR), The Alzheimer’s Association TrialMatch, Brain Health Registry (BHR), and Healthy Brains, as well as registries managed by clinical trial sites. The study was coordinated by the Alzheimer’s Therapeutic Research Institute (ATRI) at the University of Southern California (USC). Periodic updates were provided to the clinical trial sites involved in the program during the development phase. A small group of “vanguard sites” were selected first, with their study teams providing feedback on the referral process, before expanding to the total sites. Each clinical site participating in the TRC receives modest financial support for local recruitment and referral efforts, separate from their reimbursement for TRC participant visits. In 2019, the TRC-PAD program became affiliated with the Alzheimer’s Clinical Trials Consortium (ACTC) with scientific guidance provided by the ACTC Steering Committee.

### Regulatory oversight

**Figure 1.**
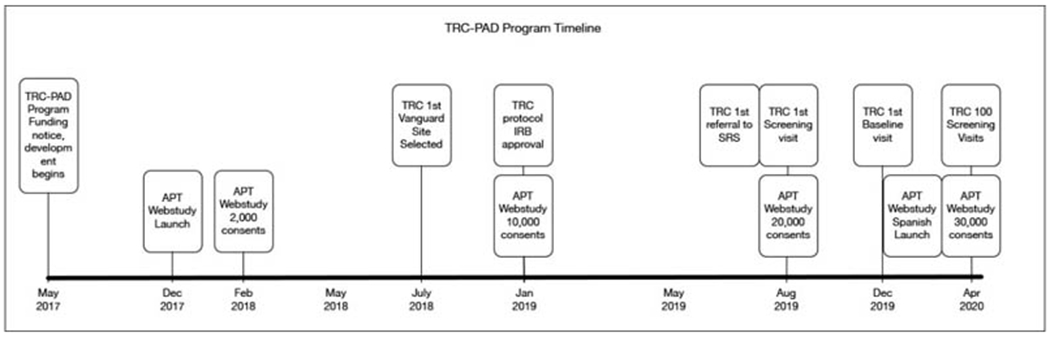
TRC-PAD Program Timeline

The APT Webstudy is overseen by the University of Southern California (USC) Institutional Review Board (IRB), which reviews and approves all participant-facing content, including the informed consent documents, web pages, emails, newsletters, and quarterly testing reminders. The IRB provided initial approval for the APT Webstudy in November 2017 and the Webstudy launched four weeks later (Figure 1). IRBs overseeing Registries also reviewed recruitment materials. The protocol describing in-person visits, screening, and enrollment in the TRC is overseen by Advarra IRB, the central IRB. In some cases, the local IRBs that oversee the clinical trial sites also required review of materials.

### APT Webstudy Participant support

Support is provided in-house by the APT Webstudy team at the USC Alzheimer’s Therapeutic Research Institute (ATRI). Participants may telephone or email the study team with their questions. Using a ticketing and tagging system, each issue is tracked centrally, which allows the support team to identify patterns and trends. Questions are triaged to subject matter experts when needed; for example, to a clinician or technical team member. Issues are reviewed centrally at regular intervals and used to improve the website and study communications.

### Retention tools

Retention of study participants and capturing longitudinal assessments, particularly cognitive testing, are critical to the program aims. The APT Webstudy team developed a participant engagement platform to optimize the Webstudy experience. Each participant is provided results of their clinical and cognitive testing over the course of the study. Reminder emails alert participants when the next quarterly assessment is due. In addition, a quarterly newsletter called “Alzheimer’s Research Today” is emailed to all participants, including updates from the field of AD research, describing upcoming studies, and providing information on new features of the Webstudy.

### APT Webstudy experience

In order to register for the APT Webstudy, participants are asked to log in using either their existing social login credentials, or to create an account by providing a username, email address and password. Once logged on, participants are considered ‘registered.’ The Webstudy is designed as a ‘walk through’ experience, with each new section opening after completion of the former section.

#### Step 1

Personal profile. Participants provide basic information including age, race and ethnicity, education, zip code, whether they have been diagnosed with Alzheimer’s disease, and whether they are interested in participating in future AD clinical trials and are willing to share information with clinical sites near them.

#### Step 2

Consent. Each participant is asked to indicate whether they agree to participate or do not agree to participate. The consent form is displayed online and may be downloaded. Consent is required to move forward and may be revoked at any time.

#### Step 3

Lifestyle. Participants are asked brief questions about diet and lifestyle. Questions about prior genetic and amyloid testing were added in January 2019, 12 months after the APT Webstudy launched. Participants enrolled prior to this question being included are prompted to respond to these questions the next time they sign on.

#### Step 4

Remote Cognitive and Functional Assessments. The Cognitive Function Instrument (CFI) is a 15-item participant-reported questionnaire (6, 7). This assessment captures the participant’s perceived ability to perform high level functional tasks in daily life, as well as their sense of overall cognitive functional ability. The participant self-reported CFI has been validated in prior studies to provide early indication of future cognitive decline (7). The Cogstate Brief Battery (CBB), comprised of four simple playing card tasks measuring psychomotor speed and recent memory (8), is used to assess cognition and memory function. The One-Card Learning Test has shown particular sensitivity to amyloid-related decline in preclinical and prodromal AD (9).

#### Step 5: Review Scores

After completing the remote assessments, the participant can review their CFI scores in a ‘Dashboard’ view. There is a description below the score of the test, explaining what the scores might mean, (e.g. “An increasing score over time might mean cognitive decline"). CBB scores are processed within 2-5 days, and participants are notified by email when scores are available. The website description of the CBB emphasizes that the tool is used for research, and that a change in score between −10 to +10 is considered normal. After completion, the cognitive test questions are no longer available to the participant, and the next available testing date is displayed (3 months from previous test date).

### Clinical Site Referrals

Data from the APT Webstudy are evaluated monthly using an adaptive algorithm. This algorithm uses statistical models to assess each participant’s risk of AD amyloidosis (5). In order to be referred, participants must have consented to the APT Webstudy, agreed to share information with researchers, and provided a valid zip code. Participants are ranked by their predicted risk, and those with the highest risk are referred to the nearest TRC-PAD site based on their zip code. Site referrals are provided via a secure web-based tool, the Site Referral System (SRS), with the flow of participants customized to meet the site’s capacity. Site staff reach out to participants using their preferred method of contact, conduct prescreening, and if the participants are interested and appears to be eligible, invites them for an in-person screening visit to confirm eligibility for the Trial-Ready Cohort (TRC).

### Trial Ready Cohort (TRC)

The eligibility criteria for TRC-PAD broadly encompass both current and upcoming clinical trials in prodromal and preclinical AD, with the aim of enrolling 2.0 participants; approximately 1,000 preclinical and 1,000 prodromal. Screening is conducted in multiple phases, first confirming clinical and cognitive eligibility and performing apolipoprotein E (APOE) genetic testing. Using this additional information, the participant’s risk assessment is updated and reviewed centrally before screening proceeds to amyloid testing, either by positron emission tomography (PET) imaging or cerebrospinal fluid (CSF) collection by lumbar puncture. Following procedures that were designed and refined for the Anti-Amyloid Treatment in Asymptomatic Alzheimer’s Disease (A4) study (10, 11), participants are told whether they are eligible for the TRC. A 21 CFR Part 11 compliant electronic data capture system was developed by the TRC-PAD study team to manage participant data (4). Broad data-sharing in the TRC consent allow the data to be potentially used as run-in data for downstream clinical trials, minimizing participant burden. Once enrolled in the TRC, participants are followed with clinical and cognitive assessments every 6 months until a clinical trial becomes available at their site. The decision to screen for a clinical trial is entirely that of the participants, with appropriate guidance from their clinician. The protocol is designed to allow participants to re-enter the cohort after participation in another study or a break for any other reason.

## Results

### APT Webstudy Enrollment

The first major increase in APT Webstudy enrollment followed an article in a San Diego newspaper in February 2018, which resulted in over 2,000 consented participants. Gradually other recruitment initiatives were rolled out, resulting in 10,000 participants in January 2019, doubling to 20,000 participants in August 2019. As of the data cut for this manuscript (April 20, 2020) there are 30,650 participants consented to the APT Webstudy. More details on APT Webstudy recruitment methods and metrics are described in another paper in this series (2).

### APT Webstudy Demographics

Participants consenting to the APT Webstudy range in age from 17 to 94 with the mean age of 64.5, and 98.8% of participants are over the age of 50. A majority (73.0%) of Webstudy participants are female. 62.2% have a parent or sibling diagnosed with AD, and 4.6% report a diagnosis of AD. 85% have post-secondary education, with 14% reporting high school or equivalent education. Participants are 92.5% Caucasian, with 2.3% Hispanic/Latino, 1.5% African American, 1.4% Asian, and 0.2% American Indian and 0.1% Pacific Islander. 53.2% of participants are retired or not working, 30.6% are working full time, and 14.7% part-time. Within the US, geographic distribution of participants is broad, with participants in 59.1% of US counties (2). About half of APT Webstudy participants report no medical concerns, with the other half most commonly reporting high blood pressure (30%), diabetes (8%), or vascular disease (4%). In terms of lifestyle, 74% exercise 1 or more hours per week, and 81% do not drink alcohol regularly (defined as 2 drinks per day or more). Most participants prefer being contacted by email (78.7%) over a phone call (3.1%).

### TRC Enrollment

**Figure 2.**
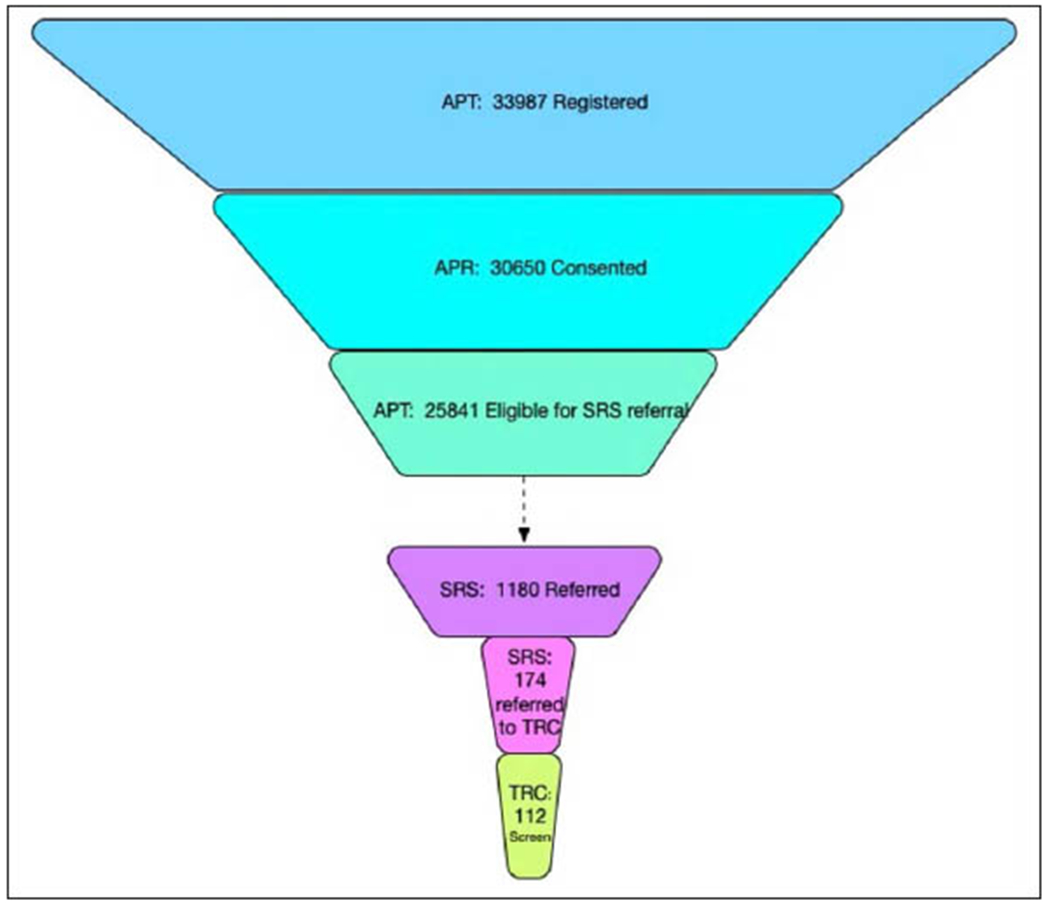
TRC-PAD Program Funnel

The first referral from SRS to a TRC-PAD Site was in July 2019 with the first screening visit conducted one month later (Figure 1). As additional sites were approved to enroll, screening activity increased to 20 screens per month in late 2019 and into early 2020. As of April 20, 2020, 1,178 participants have been referred to SRS, and 171 (14%) (Figure 2) have been subsequently referred to be screened for TRC. 112 TRC screening visits have been conducted at 9 Sites, with 54 TRC participants completing the amyloid testing, resulting in 25 participants eligible for enrollment into the TRC. 18 participants have completed a baseline visit (Figure 2).

### TRC Demographics

Of the 112 participants with an in-person screening visit, participants are aged 60-79, (mean 71.1 SD 10.6), 49.5% are women, and 93% identify as Caucasian.

### APT Webstudy Retention and Drop-outs

Participants were most likely to drop from the Webstudy at the point of consent, with 3,307 (9.7%) registering for the Webstudy but not completing the consent and 8,850 (28.9% of consented participants) not completing the initial CBB. Based on feedback from participants through the user support desk, the missed cognitive assessments are due to technical challenges and lack of compatibility of the CBB with smart phones. Retention is a challenge in the Webstudy with only 10,393 (38.2%) returning for their 2nd visit and 7,220 (28.8%) returning for visit 3. 538 participants have completed up to 8 follow up visits. The CBB retention has been lower, with 8,025 (29.5%) completing testing for the 2nd visit, and 5,777 (23%) for the 3rd visit. 461 participants have completed the CBB for up to the 8th follow up visit.

### User Support

Since launching the APT Webstudy, over 1,900 inquiries have been received from users, with a majority (78%) received by email. The most frequent reason for support (38%) is regarding the Cogstate testing. 19% of support requests are related to logging into the Webstudy, 7% are questions related to the scores for CFI or Cogstate, 7% are for non Cogstate-related technical support, and the remainder are miscellaneous support needs. Most inquiries require more than one response and took more than 2 days to resolve. Phone inquiries require an average of 20 minutes of staff time to resolve.

### Self-report of prior testing

13.03% of the APT Webstudy participants report undergoing prior APOE testing. Of these 3,989 participants with prior testing, 28% report not carrying the APOE-4 risk gene, 33% report one copy of the APOE-4 allele, and 9% reported having 2 copies. 5% reported that they carry the risk gene but do not know the details, and 23% didn’t know the results. In contrast, only 4.03% of participants had prior amyloid testing, with 2.86% having a prior PET Scan, and 1.17% a prior lumbar puncture.

### APT clinical and cognitive assessment

**Figure 3.**
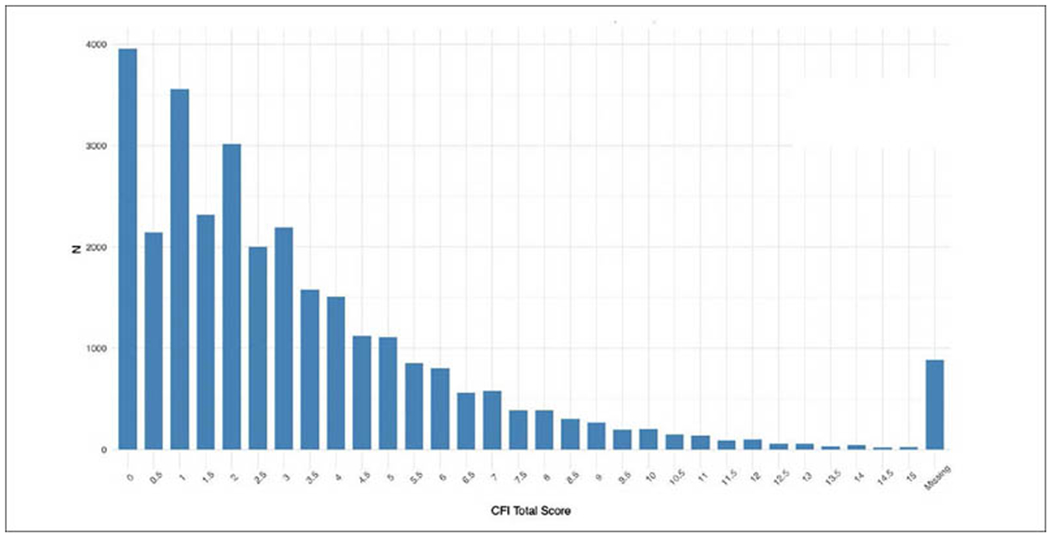
APT Webstudy Cognitive Function Instrument (CFI)

**Figure 4.**
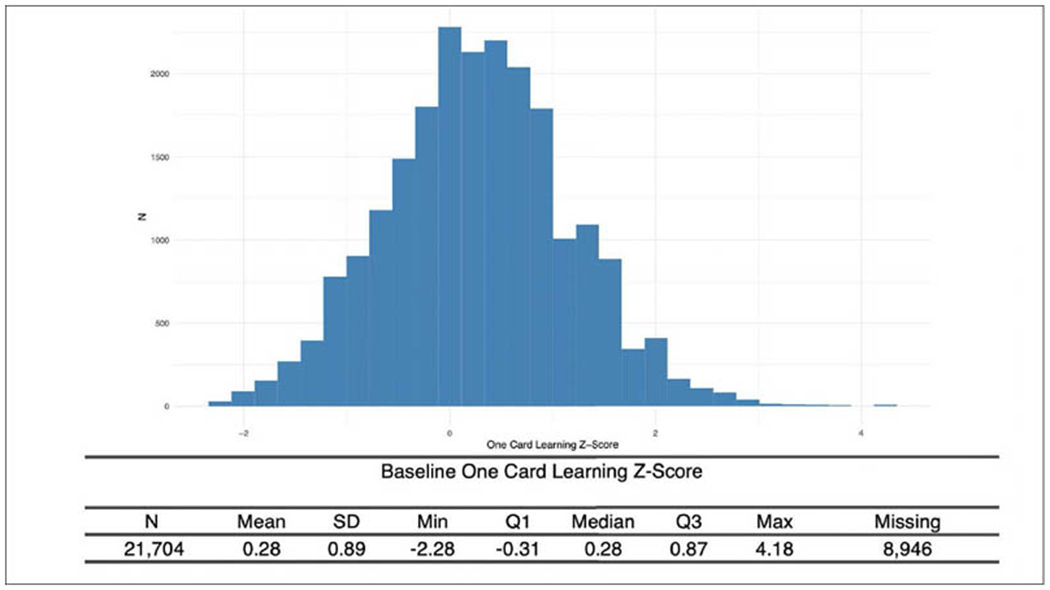
APT Webstudy Cogstate One card learning

Nearly every participant that signed consent completed the CFI (97%), with a majority scoring in normal ranges (Figure 3). 65% of Webstudy participants completed the initial Cogstate testing (Figure 4).

## Discussion

We have demonstrated that it is feasible to build a cohort of remotely-consented and enrolled participants with normal cognition, with broad geographic distribution using an unsupervised cognitive assessment battery to evaluate for increased risk for future cognitive decline. This first stage of the TRC-PAD program represents the best in what collaborative science can achieve. The partnership between the PI’s, an experienced Coordinating Center, the network of sites, academic partners, and the valuable experience and advice of investigators overseeing the APR, TrialMatch, HealthyBrains, and BHR have been critical to this success.

In general, the group of individuals enrolled in the APT Webstudy are similar to those enrolled in clinical trials, with most being highly educated and Caucasian, and a majority reporting a family history of AD. We were intentional in designing the APT assessments to be as brief as possible, and believe that low drop-out rates during initial visit is due to this. The most commonly reported problem leading to missing information on the CBB was incompatibility with smart phones; we expect that compatibility will be improved in the future.

Retention to the APT Webstudy is comparable to what has been reported by online Registry studies (12) and remains a significant challenge. Capturing longitudinal information is an important goal of TRC-PAD. More work is needed to understand why participants are not returning, in order to improve content, language, and presentation.

The APT Webstudy and TRC have both recruited a mostly white and highly educated group, which limits the representativeness of clinical trial participants using this program to the general population. We hope to improve accessibility of the APT Webstudy with the recently released Spanish translation and Spanish-language user support.

Providing consistent and knowledgeable user support for a remote Webstudy has been critical to success. We have found great value in using a centralized ticketing system, which consolidates multiple communication channels (e.g. email, telephone) and allows the study team to identify trends and prioritize development and refinement of procedures.

Ultimately, the success of TRC-PAD will be measured by efficient referral of representative participants from TRC-PAD into clinical trials. Can we predict brain amyloid elevation using Webstudy data augmented by in-person assessment, APOE genotyping and eventually plasma amyloid peptide testing (3) to reduce screening amyloid PET expenses? Can we reduce the long recruitment and screening timelines seen in studies like A4 and early symptomatic-stage AD trials? Can we minimize participant and site burden through efficient design and data-sharing between TRC-PAD and clinical trials? How do we enroll an inclusive group of individuals who are representative of the population at greatest risk for cognitive decline due to AD? TRC-PAD remains a work in progress. Continuing adjustments to its design are essential to optimizing its value.

## Figures and Tables

**Table 1.** APT and TRC Demographics (April 20, 2020)

	APT Webstudy	Trial-ready Cohort (TRC)
	Consented	
	30,650	112
	*Age at Consent*	
Below 50	357 (1.16%)	0
50-59	8,886 (28.99%)	0
60-69	13,533 (44.15%)	38 (33.9%)
70-79	6,814 (22.23%)	71 (63.4%)
80-89	967 (3.15%)	0
Over 90	39 (0.13%)	0
Missing	54 (0.18%)	2
Mean Age	64.56	71.1
	*Gender*	
Male	8,212 (26.8%)	56 (50.5%)
Female	22,389 (73.0%)	55 (49.5%)
Missing	49 (0.2%)	0
	*Race and Ethnicity*	
Asian	432 (1.4%)	3 (2.7%)
African-American	474 (1.5%)	3 (2.7%)
American Indian or Alaska Native	67 (0.2%)	0
Hawaiian/Pacific Islander	39 (0.1%)	0
Hispanic or Latino	711 (2.3%)	1 (0.9%)
White	28,363 (92.5%)	104 (93.7%)
Other, prefer not to answer	457 (1.5%)	0
Missing	107 (0.3%)	1 (0.9%)
	*Education level*	
Advanced Degree	12,018 (39.2%)	
College	14,030 (45.8%)	
High School	4,371 (14.3%)	
Prefer not to answer	136 (0.4%)	
missing	95 (0.3%)	
	*Employment Status*	
Retired/not working	16,319 (53.2%)	
Full time	9,375 (30.6%)	
Part time	4,513 (14.7%)	
Prefer not to answer	213 (0.7%)	
Missing	230 (0.8%)	
	*Do you have a diagnosis of Alzheimer’s disease?*	
Yes	1,398 (4.6%)	
No	28,999 (94.6%)	
Missing	253 (0.8%)	
	*Parent or sibling diagnosed with Alzheimer’s disease or dementia?*	
Yes	19,201 (62.6%)	
No	10,976 (35.8%)	
Prefer not to answer	247 (0.8%)	
Missing	226 (0.7%)	
	*Exercise regularly (one hour per week)*	
Yes	22,784 (74.3%)	
No	7,514 (24.5%)	
Prefer not to answer	128 (0.4%)	
Missing	224 (0.7%)	
	*Drinks alcohol regularly (2 drinks per day or more)*	
Yes	5419 (17.7%)	
No	24,789 (80.9%)	
Prefer not to answer	156 (0.5%)	
Missing	286 (0.9%)	
	*Preferred contact method*	
Email	24,145 (78.8%)	
Phone call	941 (3.1%)	
No preference	1,401 (4.6%)	
Missing	4,163 (13.6%)	
	*Medical History*	
Diabetes	2,682 (8.75%)	
High Blood Pressure	10,228 (33.47%)	
Vascular Disease	1,320 (4.31%)	
None of the above	15,192 (49.57%)	
Prefer not to answer	53 (0.17%)	
